# Monitoring cognitive load while playing exergames in a four-week intervention for older adults: an explorative EEG study

**DOI:** 10.1038/s41598-025-19183-4

**Published:** 2025-09-12

**Authors:** Helen Müller, Daniel Büchel, Nina Skjaeret-Maroni, Beatrix Vereijken, Jochen Baumeister

**Affiliations:** 1 Exercise Science & Neuroscience Unit, Department Exercise & Health, Paderborn University, Warburger Str. 100, 33098 Paderborn, Germany; 2 Department of Neuromedicine and Movement Science, Norwegian University of Science and Technology (NTNU), Trondheim, 7491 Norway

**Keywords:** Frontal theta, Motor-cognitive training, Cognition, Electroencephalography, Outcomes research, Biomarkers

## Abstract

Exergaming combines physical and cognitive components in a virtual game and has demonstrated improvements in physical and cognitive functions in older adults. However, methods to monitor cognitive demand during exergaming remain underexplored. This study investigated the sustained cognitive involvement while exergaming during a four-week intervention for older adults, utilizing EEG. The aim is to explore the impact of exergaming on changes in frontal theta power across multiple sessions throughout a four-week intervention in older adults. 21 independently living older adults (mean age 74.80 years ± 0.81; 8 females) completed a 4-week exergaming intervention where they played two different exergames (Puzzle and Fox) at two difficulty levels. Mean power spectral density in the theta band (4–7 Hz) derived from frontal brain regions at sessions 1,2,3,6,9, and 12 during exergaming. Frontal theta was significantly higher during exergaming across all games and difficulty levels compared to the reference movement (*p* < 0.01). Performance and the theta power change significantly increased over time (*p* < 0.001), indicating sustained and increasing cognitive engagement. This study is the first to repeatedly measure EEG during an exergaming intervention, confirming frontal theta activity as a robust marker of cognitive involvement. Monitoring players repeatedly during exergaming provides the basis for effective and adaptive interventions. Trial registered at DRKS (DRKS00034786).

## Introduction

As individuals age, the risks of both illness and decline in physical function increase and can contribute to cognitive impairment, frailty, and falls^1^. Physical exercise can counteract this and improve both physical and cognitive function in older adults^2^. Exergames combine physical and cognitive elements in games that require physical movement in an interactive and cognitively demanding environment^3^. Such games have been found to improve physical functions such as balance or gait in older adults^4^. Furthermore, previous studies have shown that exergames also improve cognitive function in older adults, including specific domains of executive functions, attentional processing, and visuospatial skills^5^. Therefore, exergaming is a promising approach to counteract age-related declines.

When aiming to improve cognitive functions through exercise, it is important to consider the heterogeneous outcomes across individuals when prescribing the same physical exercise^6^. As a solution, individualized training protocols which incorporate inter-individual differences in performance capacities were suggested resulting in a similar training load for all participants^6^. Over time, individualized and adaptive training protocols seem to result in larger training effects than training with constant demands^7^. For combined exercise modes such as exergaming, adaptations of both the physical and cognitive load should be considered in terms of setting adequate training stimuli. Physical load refers to the physiological and biomechanical demands imposed on the body during movement, such as cardiorespiratory or motor effort, while cognitive load describes the mental effort related to executive functions such as attention, inhibition, or working memory during task performance^8^. The physical load induced by exergames can be manipulated through adaptations of game characteristics^9–11^ and monitored through parameters such as heart rate^12^ rate of perceived exertion (RPE)^13^ accelerometer measures^9^ or movement characteristics^11^. In contrast, monitoring the cognitive load during exergaming as the non-motor complement of the exercise mode is more complex. Accordingly, most studies rely on proxy measures such as cognitive tests that reveal increased performance after an exergaming intervention^14^. However, for a tailored prescription of exergames according to individual capabilities, methods are required that provide insights into the cognitive load on individuals when exergaming. Since cognitive load relies on both the task-related complexity and the neural resources an individual recruits to accomplish a given task, both external aspects and internal parameters can be used to quantify cognitive demand^8^. On the one hand, changes in performance such as more errors or increased reaction time can point to an increased cognitive load from an external perspective. On the other hand, the application of neurophysiological methods such as electroencephalography (EEG) seems promising, since these allow for the monitoring of the neural processes in response to cognitively challenging exergames^3^ reflecting the degree of cognitive involvement.

The monitoring of brain activity as a function of cognitive involvement during dynamic exergaming requires a neurophysiological technique with high portability^15^. EEG is a mobile neurophysiological method that may describe the cognitive involvement of participants during an intervention. EEG has previously been applied to quantify sensorimotor and cognitive processes associated with different types of exercise such as balance^16^ or table tennis^17^ but also during exergaming activities^10,18^. Here, systematic modulations of electrocortical oscillations in different regions of the brain provide insights into the brain’s involvement in exercise.

Previous studies examining cognitive workload using EEG have recommended both frontal theta and parieto-occipital alpha rhythms as relevant parameters^19^. However, in the context of exergaming, alpha activity has shown a more heterogeneous and variable pattern across individuals and tasks, suggesting that the spontaneous alpha response to different exergames in parietal regions are less linear and therefore less suitable for longitudinal monitoring^10^. Therefore, frontal theta power seems more feasible to monitor cognitive workload compared to beta or alpha activity that showed more inconclusive results^20^ Frontal theta is an important indicator of cognitive involvement and information processing in both young and older adults^21–23^. Evidence suggests that frontal theta power increases during cognitive testing in young adults, and further increases with higher task difficulty^22,24^. Particularly on older adults, increased frontal activation during cognitively demanding tasks has been discussed as indicator of greater cognitive effort and compensatory engagement of executive resources to maintain performance^25^. These age-related changes in neural recruitment provide a conceptual basis for using frontal theta power as a sensitive marker of cognitive workload and adaptive control processes^20^. Assessing brain activity and motor behavior at the same time may provide complementary insights into the cognitive engagement of elderly during exergaming.

Initial studies that measured EEG while exergaming suggest that the frontal cortex of the brain is highly involved in both young^18^ and older adults^10^. Both studies observed that cognitively and physically challenging exergames demonstrate an increase in frontal theta power in comparison to similar movements without cognitive stimuli in front of a black screen^10,18^. Since frontal theta oscillations likely reflect the cognitive processes induced by exergames, it seems promising to utilize the increase in frontal theta power as a monitoring variable to quantify the cognitive involvement during exergaming interventions.

The main aim of this study was to explore the impact of exergaming on changes in frontal theta power across multiple sessions throughout a four-week intervention in a group of independently living older adults. The secondary aims were to further explore the potential effect of intervention duration and game characteristics (game type and difficulty level) on changes in frontal theta power. Therefore, an intervention design was devised wherein older adults performed two exergames in two distinct difficulty levels three times a week. EEG data was recorded at six time points during the four-week intervention while exergaming.

## Results

Assumptions of parametric testing were assessed as described in the Methods. No significant violations of normality were observed, where sphericity assumptions were violated in repeated measures ANOVA, Greenhouse–Geisser corrections were applied.

### Frontal theta power

For both games, the frontal theta power was higher while exergaming compared to the reference movement across all sessions. Statistical analysis showed a significant effect of Condition (F(2,595.06) = 4.732, *p* < 0.01) with significantly higher frontal theta power in both the easy (MD 0.578, CI 95% 0.14–1.01, *p* < 0.01) and hard (MD 0.605, CI 95% 0.17–1.04, *p* < 0.01) gaming condition compared to the reference movement. No significant difference was found when comparing the two difficulty levels (MD −0.03, CI 95% −0.46–0.41, *p* = 0.904).

**Fig. 1:**
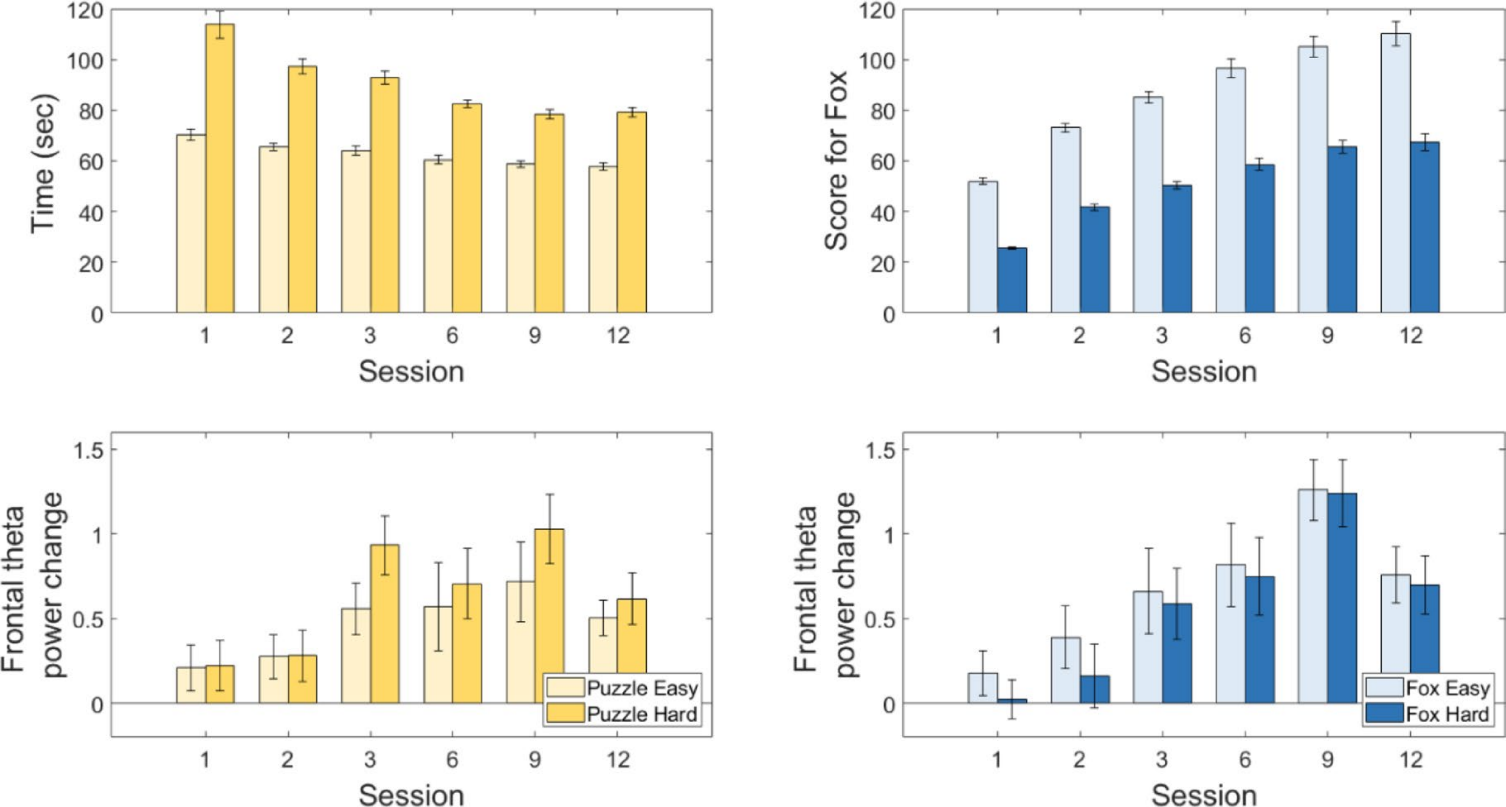
Top: Mean performance (Game Score) with SE across the 12 sessions. Better performance is indicated by a shorter playing time in the Puzzle game (yellow bars), and a higher score in the Fox game (blue bars). Bottom: Change in frontal theta from the reference movement to the corresponding games for the Puzzle game (yellow bars) and Fox game (blue bars) at both levels of difficulty. Positive values indicate an increased cognitive involvement during exergaming.

The mixed model analysis revealed a significant effect of Session (F(5,393.52) = 15.0, *p* < 0.001) on the delta of theta power, indicating that the difference in frontal theta power between the reference movement and the gaming conditions increased across the intervention. Detailed post hoc tests are shown in Table 1. There was no significant effect of Game (F(1,388.23) = 1.28, *p* = 0.26) or Difficulty Level (F(1,388.23) = 0.14, *p* = 0.71) on frontal theta power change. Furthermore, none of the interactions were significant (Game*Difficulty Level (F(1,372.23) = 3.19, *p* = 0.75), Game*Session (F(5, 372.23) = 1.16, *p* = 0.33), Difficulty Level*Session (F(5, 372.23) = 0.37, *p* = 0.87), Game*Difficulty Level*Session (F(5, 372.23) = 0.09, *p* = 0.99). The frontal theta power change across the intervention is displayed in Fig. 1.

**Table 1:** Results from significant post hoc tests of the effect of session on frontal theta power change. Mean differences between the sessions include mean values of both games (Puzzle and Fox) and difficulty levels (Easy and Hard). P-values presented in bold indicate a significant difference.

Comparison Session	Mean Difference	CI 95%	*p*-value
1–2	-0.16	-0.41 – 0.09	0.20
1–3	-0.58	-0.84 – -0.32	**< 0.001**
1–6	-0.61	-0.86 – -0.35	**< 0.001**
1–9	-0.98	-1.24 – -0.73	**< 0.001**
1–12	-0.48	-0.73 – -0.23	**< 0.001**
2–3	-0.42	-0.67 – -0.17	**0.001**
2–6	-0.45	-0.69 – -0.2	**< 0.001**
2–9	-0.82	-1.06 – -0.58	**< 0.001**
2–12	-0.32	-0.56 – -0.082	**0.009**
3–6	-0.03	-0.28 – 0.23	0.83
3–9	-0.40	-0.66 – -0.15	**0.002**
3–12	0.09	-0.16 – 0.35	0.46
6–9	-0.38	-0.62 – -0.13	**0.003**
6–12	0.12	-0.13 – 0.37	0.33
9–12	0.49	0.26 – 0.74	**< 0.001**

### Exergaming performance

The participants improved their performance significantly in both games (Puzzle: F(1.49,29.85) = 44.57, *p* < 0.001, η2 = 0.69; Fox: F(1.90,37.94) = 97.30, *p* < 0.001, η2 = 0.83). Post-hoc tests indicated that the performance increased significantly from session to session until session 6 for the Puzzle game and until session 9 for the Fox game (Table 2). Comparing the easy and hard difficulty level revealed that the performance was significantly better in the easy condition for both games (Puzzle: F(1,20) = 258.14, *p* < 0.001, η2 = 0.93; Fox: F(1,20) = 1596.21, *p* < 0.001, η2 = 0.99). Furthermore, a significant interaction effect Difficulty Level*Session was found (Puzzle: F(2.25,44.91) = 23.15, *p* < 0.001, η2 = 0.54; Fox: F(2.56,51.20) = 34.50, *p* < 0.001, η2 = 0.63), indicating that the participants increased their performance more at the hard level in the Puzzle game and at the easy level in the Fox game. The performance of the participants in the games throughout the intervention is shown in Fig. 1.

**Table 2:** Results from post hoc tests of the effect of Session on performance in Puzzle and Fox game. The repeated measures ANOVA was used to compare the mean value of both difficulty levels (Easy and Hard) separately for each game. P-values presented in bold indicate a significant difference.

Comparison Session	Puzzle			Fox		
	Mean Difference	CI 95%	p-value	Mean Difference	CI 95%	p-value
1–2	10.59	3.665 – 17.51	**< 0.001**	-19.26	-23.68 – -14.91	**< 0.001**
1–3	13.56	5.245 – 21.87	**< 0.001**	-29.54	-35.60 – -23.48	**< 0.001**
1–6	20.60	10.03 – 31.17	**< 0.001**	-39.37	-49.28 – -29.46	**< 0.001**
1–9	23.37	12.97 – 33.78	**< 0.001**	-47.04	-57.78 – -36.29	**< 0.001**
1–12	23.49	12.50 – 34.47	**< 0.001**	-50.63	-63.87 – -37.40	**< 0.001**
2–3	2.97	0.31 – 5.63	**0.02**	-10.27	-16.47 – -4.08	**< 0.001**
2–6	10.02	5.56 – 14.48	**< 0.001**	-20.11	-29.77 – -10.45	**< 0.001**
2–9	12.79	7.75 – 17.83	**< 0.001**	-27.77	-38.19 – -17.35	**< 0.001**
2–12	12.90	7.22 – 18.58	**< 0.001**	-31.37	-44.20 – -18.54	**< 0.001**
3–6	7.04	3.29 – 10.80	**< 0.001**	-9.83	-17.67 – -2.00	**0.007**
3–9	9.82	5.51 – 14.13	**< 0.001**	-17.50	-26.59 – -8.42	**< 0.001**
3–12	9.93	5.23 – 14.63	**< 0.001**	-21.10	-31.83 – -10.36	**< 0.001**
6–9	2.77	−0.29 – 5.84	0.103	-7.67	-13.57 – -1.77	**0.005**
6–12	2.88	−0.75 – 6.52	0.234	-11.26	-20.04 – -2.49	**0.006**
9–12	0.11	−2.54 – 2.77	1.000	-3.60	-8.09 – 0.90	0.223

## Discussion

The main aim of this study was to explore changes in frontal theta power across multiple sessions of exergaming throughout a four-week intervention in a group of independently living older adults. First, we observed that the frontal theta power induced by exergaming was significantly enhanced compared to the reference movement at all six investigated time points of the 4-week intervention, indicating a sustained higher activation of the frontal cortex while exergaming. In addition, we observed that this change in frontal theta power induced by exergaming increased significantly throughout the 4-week period, while game performance significantly improved over time as well. With reference to our secondary aims, no effects of game type nor difficulty were observed on changes in frontal theta activation, while game performance was significantly better in the easy condition of both games.

Across the 4 weeks of exergaming intervention, increased frontal theta power while exergaming was observed at all analyzed time points. An increase in frontal theta power is widely recognized as a marker of cognitive processing, indicating that exergames were cognitively demanding for the older adults at all time points, also after repeated exergaming sessions for 4 weeks. These findings are in line with and further extend previous cross-sectional studies that observed modulations of prefrontal cortical involvement^26–28^ and increases in frontal theta activity during single exergaming sessions^10,18^. Previous research has linked frontal theta to the anterior cingulate cortex (ACC)^21,29,30^, a crucial component of the human attentional system^31,32^. Accordingly, studies describe frontal theta activity as a prominent indicator of cognitive demand and information processing in young^21,22^ and older^23^ adults. Moreover, frontal theta power is linked to attentional processes during cognitive^24,33^ and sensorimotor^16,34^ tasks. The increase in frontal theta activity in the current study therefore indicates that exergames remain a cognitively demanding exercise mode for older adults despite repeated sessions. Taken together, changes in frontal theta power might serve as a measure to monitor the cognitive involvement of exergaming, what is consistent with previous findings of EEG power spectral measures^20^. This may enable the control of an exergaming intervention based on both physical and cognitive characteristics.

To the best of our knowledge, no previous study monitored and described changes in frontal theta power repeatedly throughout an exercise intervention. Previous studies typically measured cognitive performance before and after the intervention^35^ but not throughout an intervention. Therefore, the current study is the first to show that even simple mini-games, played repeatedly for 12 sessions over a period of 4 weeks in a standardized environment, can increase the participants’ cognitive involvement in every single session. Therefore, the present results illustrate that cognitive involvement during exergames does not necessarily disappear due to a habituation effect and may persist over time, even after four weeks of repeating the same exercise.

As discussed in the previous section, frontal theta power was enhanced compared to the reference movement at all time points for all exergaming conditions. Furthermore, the difference between reference movement and exergaming conditions as a function of cognitive involvement increased throughout the intervention. The increasing change in frontal theta power over time was in line with improved game performance and could be interpreted as an indicator of increased recruitment of attentional resources throughout the intervention.

Comparisons of frontal theta activity between novices and experts in earlier cross-sectional studies revealed that experts exhibit higher frontal theta than novices during goal-directed sports movements such as shooting^36–38^ or golf putting^39^. This observation has been discussed as a marker of higher focused attention in experts^36,39^. Transferred to this study, older individuals may become more familiar with the task after repeated exergaming sessions due to the automatization of the movement and control of the game. Thus, they might be able to focus their attention more precisely on critical details of the game which could in turn affect their performance positively. The increase in exergaming-induced change in frontal theta power over time may potentially indicate adaptations to exergaming training in terms of more focused and automatized playing. Simultaneously, the participants’ performance in both games increased throughout the intervention, with the most improvement occurring until session six and leveling off afterwards. This is a typical pattern observed in motor learning studies^40^ as well as in exergaming studies^41,42^. The leveling off can be interpreted as a sign of mastery, indicating that the participants were able to play the games sufficiently. Taken together, a 4-week exergaming intervention may lead to adaptations such as automatization and improved focusing of playing. These adaptations might be reflected by improved exergaming performance on the one hand and increased cognitive involvement on the other hand.

In the present study, we further aimed to monitor cognitive involvement due to repeated assessment of frontal theta power during different exergames with varying levels of difficulty. In line with results from earlier cross-sectional examinations^10^our results did not find an effect of difficulty level on frontal theta power in older adults. However, there was an effect of difficulty level on performance in that performance was significantly better in the easy condition compared to the hard version, indicating that the harder level was indeed more demanding. These findings are in line with findings from studies examining coordination^43^ and exergaming tasks^10^ in older adults where increased difficulty level affects the performance but is not necessarily accompanied by increased theta power. However, the findings stand in contrast to observations in young participants, where increased difficulty is associated with increased theta power in cognitive tasks^22,24^ and exergaming^18^. According to the compensation-related utilization of neural circuits hypothesis (CRUNCH)^25^, older brains require larger effort to achieve comparable results than young brains and thus older adults may require more neural resources in similar tasks than younger people. If demands increase further, young brains are able to increase neural processing while the neural resources of older adults may reach a limit and cannot be further increased, resulting in inadequate processing and poorer outcomes^25^. In the current study, frontal activity did not increase further in the harder conditions, but the performance decreased in both games with increased difficulty level. Accordingly, it could be speculated that the older participants experienced substantial cognitive demand already in the easy condition, so that no further increases in cognitive involvement were observed during the difficult condition.

Furthermore, the participants’ frontal brain activity was not affected by the two game types in this study. Both games demonstrated increased frontal theta activity while exergaming compared to the reference movement. Therefore, neither the content of the game nor the different types of movement (leaning versus stepping) altered cognitive involvement as indexed by frontal theta power. It might be speculated that the increase in frontal theta activity compared to the reference movement results from the overall demands of exergaming, representing an interactive motor-cognitive task requiring complex information processing.

The current study reveals the first insights into changes in brain activity during an exergaming intervention. It is the first study to measure EEG repeatedly throughout a training intervention. This approach enables monitoring cognitive load repeatedly throughout the intervention instead of evaluating the effect of an intervention by measuring cognitive function only before and after the intervention. The portability of the EEG allowed for the collection of data while playing the actual game, while other modalities are limited to resting state data recorded after an exergame session. Therefore, the present study may provide information about the actual cognitive involvement while exergaming, that may complement previous findings on the effect of exergaming on cognitive functions in traditional pre-post designs.

However, measuring EEG repeatedly is associated with some methodological limitations as well. Despite counteracting the limited spatial resolution of EEG^15^ with independent component analysis and individual head model coordinates collected using Captrack, the dipole locations are estimated and do not necessarily represent the actual cortical source of the signal^44^. Furthermore, the localization of the frontal clusters differed slightly across the sessions, which may influence the absolute frontal theta activity as the sources of the brain signals might differ slightly.

The EEG data was analyzed condition-averaged, including both moments with decision-making and moments with less cognitive involvement, making it challenging to establish whether theta activity represents specific control-related processes^45,46^. Future studies could include an event-related analysis in addition to zoom in on the short periods of time around the stimuli that induce cognitive involvement^47^. However, such an approach requires a high number of events, which could lead to fatigue and potential adverse effects in the participants, especially in older adults.

This is the first study to measure and describe EEG outcomes repeatedly during an exergaming intervention while older adults were exergaming. The findings indicate that exergaming inherently involves cognitive demands despite differences in game type or difficulty level, and that such demands persist over the course of a 4-week intervention and are not lost due to e.g. habituation. Exergames can thus be effective in engaging cognitive abilities over an extended exercise period. Moreover, the intervention led to increased frontal theta activity while exergaming compared to the reference movement, highlighting its positive impact. In sum, we provided the first evidence that exergames address the cognitive demands during an intervention period and thus might be effective in training cognitive functions in older adults. However, further research is necessary to interpret the implications of frontal theta while exergaming.

## Methods

### Study design and setting

A one-group multiple posttest design was conducted spanning four weeks and consisting of six measurement points. Ethical approval for this study was obtained from the Ethics Committee of Paderborn University, and the research adhered to the Declaration of Helsinki. The study was registered at the German Clinical Trials Register (DRKS00034786, date of registration: 30/07/2024, retrospectively registered).

All assessments were conducted at the laboratory of the Exercise Science and Neuroscience Unit, Paderborn University, Germany between August 2019 and April 2020.

### Participants

Participants were recruited through local newspaper advertisements and word-of-mouth referrals. During an information session, individuals expressing interest underwent comprehensive screening for inclusion and exclusion criteria. Inclusion criteria were: (1) independently living older adults aged 70 years or older, and (2) no prior experience in exergaming. The term “independently living” was used to reflect sufficient mobility, cognitive function, and daily functional abilities to safely and meaningfully participate in the intervention and included all older adults living in the community without substantial assistance in daily activities. Exclusion criteria were a history of neurodegenerative or neurologic diseases, acute physical or mental problems precluding safe exergaming participation for 4 weeks, or any surgical or injury-related conditions affecting pain-free movement during the study. During this information session, individuals also had the opportunity to familiarize themselves with the equipment and laboratory environment before the data collection. Eligible participants provided written informed consent and were subsequently scheduled for training sessions. In total, 28 older adults (mean age 74.57 ± 0.78 years, mean height 172.04 ± 1.86 cm, mean weight 76.85 ± 2.26 kg, 14 females) participated in the study. All participants completed every training session. Due to limited EEG data quality, 7 participants were excluded from the analyses. The final 21 participants (8 females) were 74.80 years old (range 70–84) with normal to good physical abilities for their age according to the Community Balance and Mobility Scale (CBMS)^48^ largely age-appropriate cognitive performance with no indication of manifest cognitive impairment based on the Montreal Cognitive Assessment (MoCa)^49^ and low fear of falling according to the Falls Efficacy Scale International (FES-I)^50^. Detailed information about the participants is provided in Table 3.

**Table 3:** Participant information for those who were included in the analysis (*n* = 21, 8 females). Age, height, weight, cognitive function (MoCa – Montreal cognitive Assessment), physical function (CBMS – Community Balance and Mobility Scale), and fear of falling (FES-I – Falls Efficacy Scale) were accessed before the intervention at the initial session.

Variable	Mean	SE	Min	Max
Age (years)	74.80	0.81	70	84
Height (cm)	174.20	1.71	158	192
Weight (kg)	80.10	2.09	58	102
MoCA	25	0.33	21	28
CBMS	64.52	2.24	37	86
FES-I	18.02	0.32	16	22

### Exergaming system

**Fig. 2:**
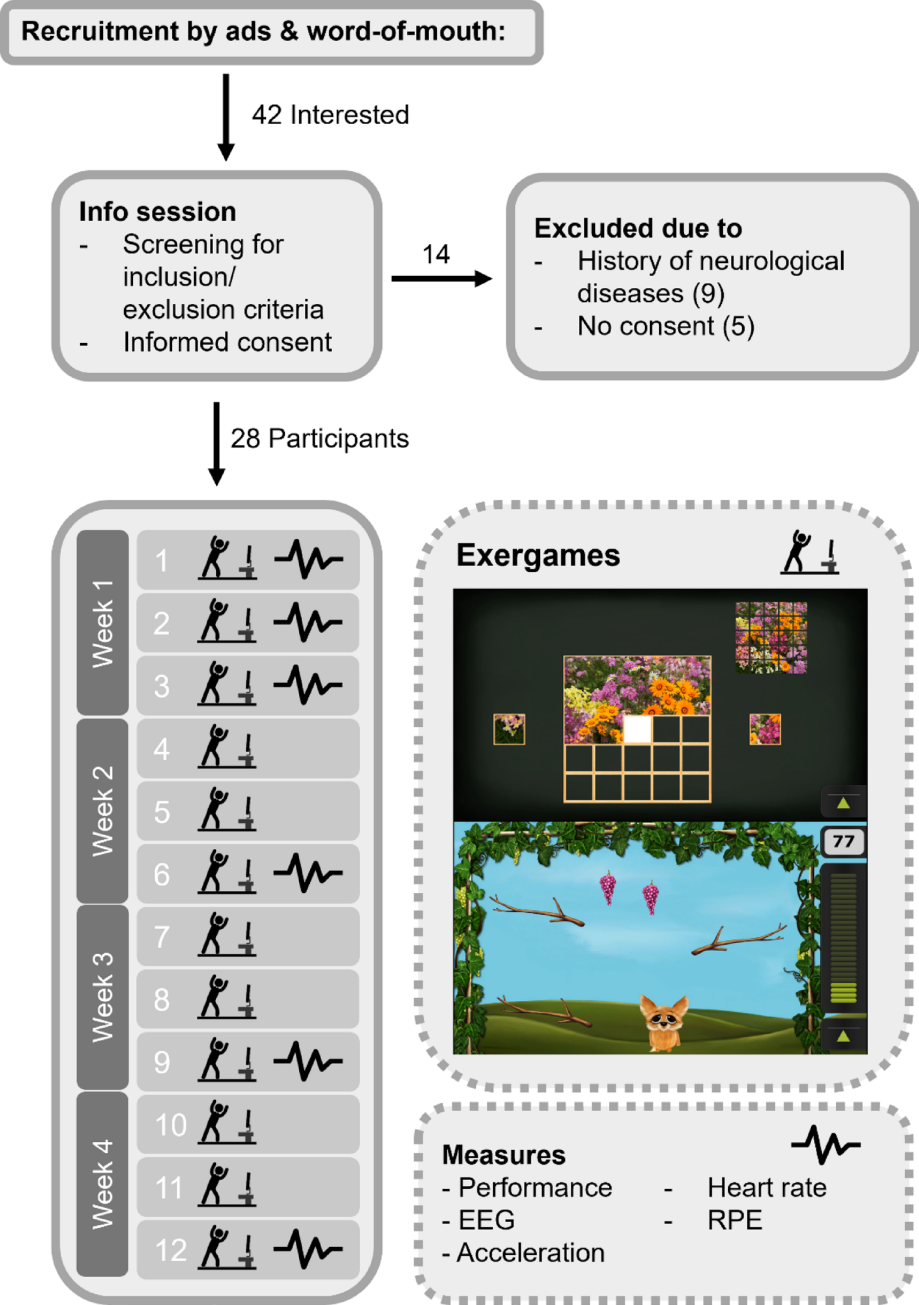
Flowchart of the study design. Prior to the exergaming intervention, all participants were invited to an info session. 28 participants started the intervention that consisted of 12 training sessions where two exergames were played at each session. Top: Screenshot of the Puzzle game, Bottom: Screenshot of the Fox Game. Furthermore, in session 1,2,3,6,9, and 12 performance, EEG, acceleration, heart rate and RPE were recorded.

Two exergames at varying difficulty levels were selected from the Silverfit System (SilverFit BV 3D, Woerden, Netherlands). This system utilizes a TV screen and a time-of-flight camera to record players’ body movements in three dimensions within a 5 × 5 m game area, corresponding to a 176 × 144 pixel array. The two exergames used in the intervention were selected to represent distinct categories of motor interaction^51^. The Puzzle game aligns with balance training, involving feet-in-place postural control via center-of-mass shifts. The Fox game, in contrast, represents step training, requiring lateral stepping for navigation, emphasizing coordinated weight transfer and dynamic balance. This allowed investigation of cognitive involvement across different motor demands. Figure 2 shows screenshots of the games.

In the Puzzle game, players control the game by leaning their upper body sideways without taking steps. The objective is to solve a 5 × 5 pieces jigsaw puzzle. In the Easy condition, a single puzzle piece appears either on the left or right side, and players select this piece by leaning in the corresponding direction. In the Hard condition, two puzzle pieces appear on each side, requiring players to select the correct piece by leaning in the corresponding direction.

In the Fox game, players navigate a fox avatar on the screen by stepping left and right. In the Easy condition, grapes fall from the top of the screen, and players aim to catch as many grapes as possible by positioning the fox avatar directly underneath. In the Hard condition, branches fall in addition and have to be avoided. At session 1, all participants played the Fox game at the same game speed level (3 out of 10), with faster game speed levels resulting in more grapes falling at a faster rate. Across the intervention, the game speed level was adjusted individually based on a combination of players’ subjective evaluations and their objective performance. After each Fox game session, participants rated the game speed level as too slow, too fast, or appropriate. If a participant perceived the game as too slow or made no mistakes (all possible grapes caught without hitting branches), the game speed level was increased in the following session. Conversely, if participants deemed the game speed too high, it was reduced. This procedure allowed individual tailoring of the game speed level throughout the exergaming intervention.

Game scores were directly exported from the Silverfit System and served as a performance metric. In the Puzzle game, the outcome measure was the time required to complete one image. For each Puzzle condition, the times for completing the seven images were averaged. In the Fox game, the game score represented the number of grapes caught, with a deduction of 2 points for each branch collision in the Hard condition. The mean of the two Fox game repetitions was calculated as the game score per condition (Easy and Hard) for each session.

### Procedure

The study protocol consisted of 12 training sessions spread across 4 weeks, with 3 sessions conducted each week (see Fig. 2). All training sessions were conducted individually for each participant under the supervision of the first author. Each training session had an approximate duration of 45 min and encompassed two different exergames (Puzzle and Fox, SilverFit BV 3D, Silverfit, Woerden, the Netherlands), each performed at two difficulty levels (Easy and Hard). The Puzzle game consisted of seven different images at both Easy and Hard difficulty levels. One Puzzle game lasted approximately between one and two minutes, depending on the performance of the participants. The Fox game was played twice at each of the two difficulty levels, with each repetition lasting 4 min. The order of these four conditions was systematically counterbalanced across participants and sessions. Between the game segments, rest periods of about 30 s were provided. To play the Puzzle game, participants leaned their upper body to the left and the right while keeping their feet in place, for the Fox game they took steps to the left and right in a range of about 5 m. Before each exergame, participants performed the corresponding movements as a reference movement for each game while looking at a black screen once each session. Each movement was demonstrated once, speed and amplitude were not controlled.

Across the four week intervention, several outcomes of interest were monitored during sessions 1, 2, 3, 6, 9, and 12. To this end, participants were equipped with a heart rate belt (Polar H10 & Polar M430, Polar Electronics, Kempele, Finland), accelerometers (AX3, Axivity, Newcastle upon Tyne, United Kingdom) fixed to their lower back and feet, and a 64-channel EEG system (Live Amp 64, Brain Products, Gilching, Germany) before each of the 6 sessions. After each exergame, the participants rated their perceived exertion for each game. Changes in physical function (CBMS^48^, and fear of falling (FES-I^50^, were assessed in the initial and final sessions. The present paper focuses on the analysis of the EEG data, the other data was published elsewhere^10,52^.

### EEG data acquisition and processing

Continuous recordings of brain activity throughout the sessions were achieved employing 64 active EEG electrodes (ActiCap, Brain Products, Gilching, Germany) and a wireless amplifier (Live Amp 64, Brain Products, Gilching, Germany). The sampling rate was set at 500 Hz. Electrode placement followed the international 10–20 system^53^ with the ground electrode situated at the mid-forehead^54^. Online referencing was performed to FCz. Electrical impedances were lowered to < 5 kOhm by inserting gel into the space between scalp and electrode to improve the signal-to-noise ratio. For standardization, electrode positions were recorded and stored using the Cap Trak system (Brain Products, Gilching, Germany). Using a hand-held scanner, the 3D coordinates of the electrode positions on the subject’s head were tracked by comparing each electrode’s built-in LED position with the position of three anatomical landmarks: the nasion, right preauricular point, and left preauricular point, which were marked with LED lights. Participants were briefed on potential sources of EEG artifacts caused by for example blinking, teeth clenching, or excessive upper-body muscle tension, and were asked to minimize non-essential movements unrelated to gameplay during data collection.

During the study, 168 datasets from 28 participants were recorded. One dataset was excluded due to technical problems during recording, and 6 datasets were missing a condition due to lost markers identifying the start and stop of the games. Thus, 161 datasets were included in the analyses.

All EEG data processing was conducted using the EEGLAB toolbox v2020_0 ^55^ in MATLAB (Version R2019b, Mathworks Inc., Natick, United States). An established EEG processing pipeline was applied, previously utilized in studies (e.g^10,18,56^. This included the utilization of the Cleanline plug-in for sinusoidal noise removal^57^. Subsequently, the data was filtered using a FIR filter between 3 Hz and 30 Hz, referenced to a common average and downsampled to 256 Hz. Channels connected through electrical bridges due to low impedance were identified and removed using the eBridge plugin^58^. Additional noisy channels were detected and removed using the EEGLAB pop_rejchan function with a threshold of 5 SDs. On average 61.20 (SE 0.47) of 65 channels were kept per participant. Further data cleaning involved the application of the clean_rawdata EEGLAB plugin^59^. Any channels containing non-stereotypical artifacts or significant noise were eliminated. Large-amplitude artifact transients were interpolated using automated subspace reconstruction (ASR), calibrated on a clean segment of the data, with a cutoff value of 7 SDs as based on prior studies^34,60^.

The cleaned EEG signal was decomposed into independent components (ICs) using AMICA^61^. Each IC was associated with corresponding dipoles, determined using the DIPFIT toolbox^62^ and classified into brain signals or non-brain signals (muscle activity, eye activity, EKG, line noise, channel noise, other) utilizing the ICLabel plug-in^63^. Only ICs with a probability of being a brain component greater than 90%, located within cortical layers, and a residual variance (RV) < 15%, were retained for further analysis. The decomposition of the remaining channels resulted on average in 21.05 (SE 0.42) functional brain components per participant, 12.25 (SE 0.39) labeled as muscle activity, 3.02 (SE 0.07) as eye movements, 1.86 (SE 0.11) as EKG, none as line noise, 4.09 (SE 0.22) as channel noise, and 18.92 (SE 0.47) as other.

**Fig. 3:**
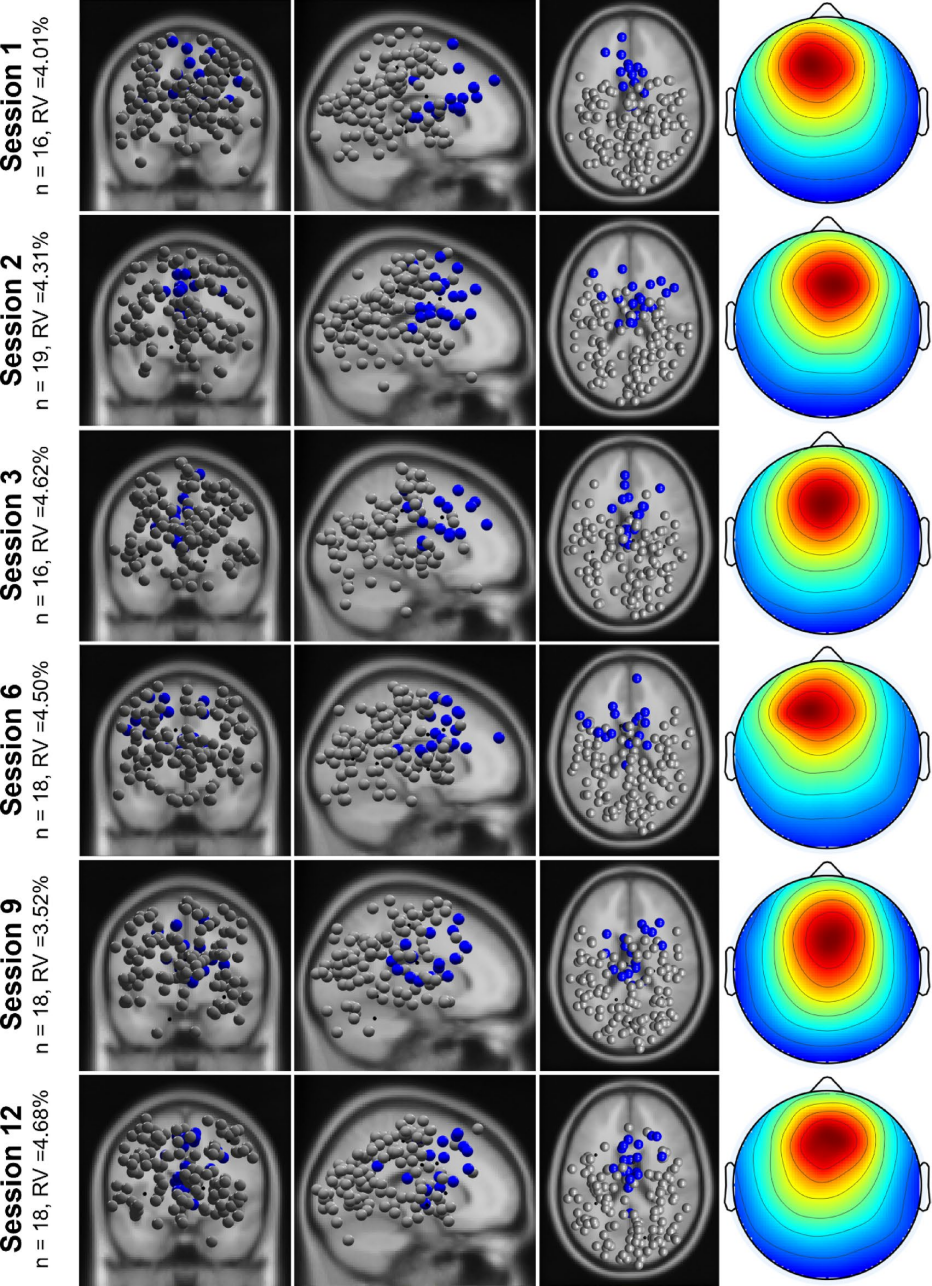
Dipoles from coronal, sagittal and top view (left) and scalp maps (right) of the frontal cluster for each session. Number of participants included in each cluster (n) and mean residual variance (RV) per cluster are presented.

For the source-based approach, the brain ICs were assigned to five clusters based on spatial (dipole location and orientation, scalp maps) and temporal (power spectra) information for each session separately. Dipoles deviating more than 3 SDs from the mean dipole were categorized as outliers. On average, each cluster included 27.73 (SE 1.77) ICs from 18.87 (SE 0.77) individuals. Due to the focus on frontal theta power as major outcome for cognitive processing, only frontal clusters were considered for further analyses. If participants contributed with more than one IC to this cluster, the IC with the most frontal dipole location was used for further analysis. The frontal clusters for each of the six analyzed sessions are presented in Fig. 3.

To investigate changes in frontal theta power, the area under the curve^54^ in the theta frequency band (4–7 Hz) was computed for each condition. Spectral power for each frequency bin (1 Hz) per component in each condition and session was exported to Excel, and the mean spectral power across all included frequencies was calculated subsequently. Participants that were missing data from 4 or more sessions were excluded from further analysis (*n* = 7). Table 4 shows the number of frontal brain ICs per participant per session. To take inter-individual differences in theta power into account, the primary dependent variable frontal theta power change was calculated as a delta score by subtracting the theta power during the associated reference measure from the theta power during the respective exergame (e.g. ΔPuzzle Easy = Puzzle Easy-Puzzle Reference). Accordingly, positive delta values indicate an increased activation of the frontal cortex.

**Table 4:** Number of functional brain ICs included into the frontal cluster per participant. If participants added more than one IC to the frontal cluster, the most frontal IC was used for further analysis. Participants 4,8,12,16,28,34,39, presented in italic, contributed only at two sessions or less frontal ICs and were excluded from further analysis.

ID	*n* ICs Session 1	*n* ICs Session 2	*n* ICs Session 3	*n* ICs Session 6	*n* ICs Session 9	*n* ICs Session 12	mean *n* Ics per Session	*n* Sessions with ICs
1	1	1	1	2	0	1	1	5
*4*	0	0	1	2	1	0	0,67	*2*
5	1	3	2	1	2	2	1,83	6
7	0	1	1	1	1	1	0,83	5
*8*	0	0	1	0	1	0	0,33	*2*
9	1	2	2	1	1	1	1,33	6
11	1	1	2	0	1	1	1,00	5
*12*	0	1	0	0	0	1	0,33	*2*
14	3	2	1	2	1	1	1,67	6
*16*	0	0	0	0	0	0	0,00	*0*
17	0	2	2	2	3	2	1,83	5
19	1	2	0	1	0	2	1,00	4
21	0	0	1	2	1	0	0,67	3
22	1	1	2	1	0	0	0,83	4
23	1	3	0	1	2	2	1,50	5
24	0	1	1	0	1	1	0,67	4
26	1	1	2	1	1	2	1,33	6
27	1	0	0	0	1	1	0,50	3
*28*	0	0	1	0	0	0	0,17	*1*
29	2	2	1	1	1	2	1,50	6
30	2	1	1	1	1	1	1,17	6
31	2	1	1	1	1	1	1,17	6
*34*	0	0	0	0	0	0	0,00	*0*
36	0	1	0	1	1	1	0,67	4
*39*	0	0	1	0	0	1	0,33	*2*
40	2	2	0	0	1	0	0,83	3
41	2	1	1	2	2	1	1,50	6
42	1	2	2	1	2	1	1,50	6
mean	0,82	1,11	0,96	0,86	0,93	0,93	0,93	3,96

### Statistical analysis

Participant characteristics and secondary data (age, height, weight, MoCA, CBMS, FES-I) were subjected to descriptive analysis. Data normality was assessed through the Shapiro-Wilk test, QQ plots, and histograms.

A linear mixed model was employed to investigate changes in mean frontal theta activity and frontal theta change over the 4-week intervention period, and/or across game type and/or difficulty level. In the model, the subject was defined as random factor to account for dependencies in the repeated measurements (random intercept model). Condition (Reference, Easy, Hard), Game (Puzzle, Fox) and Session (six occasions) were included as fixed effect factors. To simplify the model and reduce the number of parameters, Session was treated as a continuous variable (range 1–12).

To assess whether the changes during the 4-weeks intervention period varied by difficulty level, an interaction term between difficulty level and session was added to the model, incorporating two additional parameters. Furthermore, potential differences in the change across the two games (Puzzle and Fox) were evaluated through combined analyses, with potential heterogeneity by game type formally tested by including an interaction term between session and game type. Post-hoc comparisons were done using Bonferroni-correction.

Performance (game score) was analyzed using one repeated measures ANOVA per exergame with the factors Difficulty Level (2 levels) and Session (6 levels). Mauchly’s test of Sphericity was evaluated and if necessary, Greenhouse-Geisser correction was applied. Post-hoc comparisons were done using Bonferroni-correction, partial eta squared (part. η2) is reported as effect size.

The significance level for all tests was set at *p* < 0.05. All outcome measures are presented as means with standard error (± SE). All statistical analyses were conducted using IBM SPSS Statistics (version 29, IBM, Armonk, USA).

## Data Availability

The datasets generated during and/or analyzed during the current study are available from the corresponding author on reasonable request.
